# Catabolite and Oxygen Regulation of Enterohemorrhagic *Escherichia coli* Virulence

**DOI:** 10.1128/mBio.01852-16

**Published:** 2016-11-22

**Authors:** Kimberly M. Carlson-Banning, Vanessa Sperandio

**Affiliations:** Departments of Microbiology and Biochemistry, University of Texas Southwestern Medical Center, Dallas, Texas, USA

## Abstract

The biogeography of the gut is diverse in its longitudinal axis, as well as within specific microenvironments. Differential oxygenation and nutrient composition drive the membership of microbial communities in these habitats. Moreover, enteric pathogens can orchestrate further modifications to gain a competitive advantage toward host colonization. These pathogens are versatile and adept when exploiting the human colon. They expertly navigate complex environmental cues and interkingdom signaling to colonize and infect their hosts. Here we demonstrate how enterohemorrhagic *Escherichia coli* (EHEC) uses three sugar-sensing transcription factors, Cra, KdpE, and FusR, to exquisitely regulate the expression of virulence factors associated with its type III secretion system (T3SS) when exposed to various oxygen concentrations. We also explored the effect of mucin-derived nonpreferred carbon sources on EHEC growth and expression of virulence genes. Taken together, the results show that EHEC represses the expression of its T3SS when oxygen is absent, mimicking the largely anaerobic lumen, and activates its T3SS when oxygen is available through Cra. In addition, when EHEC senses mucin-derived sugars heavily present in the O-linked and N-linked glycans of the large intestine, virulence gene expression is initiated. Sugars derived from pectin, a complex plant polysaccharide digested in the large intestine, also increased virulence gene expression. Not only does EHEC sense host- and microbiota-derived interkingdom signals, it also uses oxygen availability and mucin-derived sugars liberated by the microbiota to stimulate expression of the T3SS. This precision in gene regulation allows EHEC to be an efficient pathogen with an extremely low infectious dose.

## IMPORTANCE

Enteric pathogens have to be crafty when interpreting multiple environmental cues to successfully establish themselves within complex and diverse gut microenvironments. Differences in oxygen tension and nutrient composition determine the biogeography of the gut microbiota and provide unique niches that can be exploited by enteric pathogens. EHEC is an enteric pathogen that colonizes the colon and causes outbreaks of bloody diarrhea and hemolytic-uremic syndrome worldwide. It has a very low infectious dose, which requires it to be an extremely effective pathogen. Hence, here we show that EHEC senses multiple sugar sources and oxygen levels to optimally control the expression of its virulence repertoire. This exquisite regulatory control equips EHEC to sense different intestinal compartments to colonize the host.

## INTRODUCTION

The gastrointestinal (GI) tract is a complex and diverse ecosystem populated by characteristic microbial communities within different microhabitats. Bacterial metabolism and oxygen availability play key roles in the localization and composition of these communities (1). Moreover, infection by enteric pathogens can change the environment landscape to favor pathogen expansion (2–8). Successful establishment of an enteric pathogen within a GI tract already heavily colonized by the microbiota relies on how aggressively it acquires nutrients and senses chemical signals (9). Intestinal pathogens have to precisely coordinate the expression of virulence factors. The ability to sense which nutrients are available allows bacteria to determine their location within the GI tract (10, 11). The GI mucus layer is composed of mucins—glycoproteins consisting of 80% carbohydrates (10, 11). Mucins act as GI tract signposts, as specific mucins are located along the gut (10–12). The mucin sugars released by the microbiota producing different glycosidases provide a singular nutrient environment. Therefore, coupling the expression of virulence genes with nutrient availability is one way pathogens precisely control when and where they deploy the optimal expression of their virulence repertoire to colonize the host.

Enterohemorrhagic *Escherichia coli* (EHEC) colonizes the human colon and is transmitted through contaminated food and water (13). Because of its low infectious dose (<100 cells), EHEC is a serious public health concern. Clinical symptoms range from watery, bloody diarrhea to the often fatal hemolytic-uremic syndrome (HUS) (14). Its virulence armamentarium includes the locus of enterocyte effacement (LEE), which is a pathogenicity island harboring 41 genes that are organized into five major operons, *LEE1* to *LEE5* (15–17). Encoded by these operons are a type III secretion system (T3SS) (18), an adhesin (intimin) (19) and its receptor (Tir) (20), effector proteins (21–25), and the master regulator of the LEE genes, Ler (14, 17, 26). The LEE genes are needed for EHEC to colonize the gut, as expression of the LEE genes leads to the formation of attaching and effacing (AE) lesions on enterocytes. These AE lesions are responsible for the dynamic remodeling of the host’s cytoskeleton to form pedestal-like structures beneath the bacteria (27–31).

The expression of the LEE genes is regulated by interkingdom chemical signaling involving host hormones (epinephrine and norepinephrine) and fucose (32–34). The hormone signals are sensed by bacterial adrenergic receptors, QseC (35) and QseE (36), while FusK senses fucose (33). QseC, QseE, and FusK are histidine sensor kinases (HKs). Upon sensing their respective signals, these HKs undergo autophosphorylation to initiate the phosphorylation of specific response regulators (RRs). QseC and QseE can both phosphorylate the RR QseF; however, QseC can also phosphorylate QseB and KdpE (36–38). FusK has one demonstrated RR, FusR (33). These two-component systems are interconnected, as QseB and QseF repress the transcription of *fusK* and *fusR* (33).

The cross-talk among QseC, QseE, and FusK is important, because they differentially regulate LEE gene expression. Upon QseC phosphorylation of KdpE, KdpE, in conjunction with Cra (a global regulator of genes involved in carbon metabolism [39]), activates the expression of all of the LEE genes by directly binding to the *ler* promoter under gluconeogenic conditions (34, 38, 40). Under glycolytic conditions, KdpE and Cra do not activate LEE gene expression (34). Conversely, FusR represses LEE gene transcription upon sensing fucose (33). Sensing of sugar concentrations associated with mucin-derived sugars is essential for EHEC to promote virulence, with *fusK*, *kdpE*, and *cra* mutants being attenuated for mammalian infection (7, 33, 41). EHEC senses when sugars are more abundant in the lumen or less abundant closer to enterocytes, where the glycophagic microbiota is absent, creating a gluconeogenic environment with more host hormones present. Detecting the alterations in sugar concentrations allows EHEC a way to avoid the premature expression of LEE genes, which should only be expressed at the interface with enterocytes.

In this study, our goal was to determine how much cross-talk exists among Cra, KdpE, and FusR, given their differential roles in the regulation of LEE gene expression. In addition, as oxygen availability is also a key regulator of metabolism, we studied how oxygen availability affects the regulation of LEE gene expression by Cra, KdpE, and FusR. We also evaluated the roles of different mucin-derived sugars in EHEC growth and the secretion of LEE-encoded T3SS-secreted EspB under limited-oxygen conditions.

## RESULTS

### Relationships among Cra, KdpE, and FusR in growth and virulence.

The transcription factors Cra, KdpE, and FusR directly bind to the *ler* regulatory region to control LEE gene expression (33, 34). To address the interplay among these transcription factors in the regulation of the LEE, we constructed Δ*kdpE*, Δ*kdpE* Δ*cra*, Δ*fusR* Δ*cra*, Δ*fusR* Δ*kdpE*, and Δ*fusR* Δ*kdpE* Δ*cra* EHEC deletion strains. EHEC with a single deletion of *cra*, *kdpE*, or *fusR* alone exhibits no growth defects, as previously reported (33, 34). However, deleting these genes in combination could impair bacterial growth. To determine whether the double and triple mutant strains were comparable to the wild type (WT) in growth, the constructed deletion strains were grown in either LB or 0.1% glucose with 1 mM pyruvate Dulbecco’s modified Eagle’s medium (DMEM). None of the deletion strains had growth defects compared to WT EHEC (see Fig. S1 in the supplemental material).

**FIG 1:**
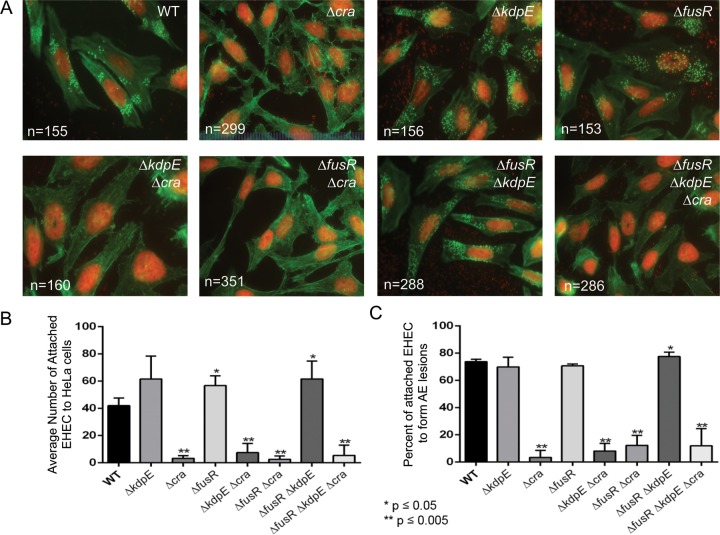
AE lesion formation in deletion strains. (A) All DNA is stained red (bacteria and HeLa cell nuclei). AE lesions are green (actin) cups beneath red bacteria. The number of quantified HeLa cells is indicated. (B) Quantification of the average number of bacteria attached per HeLa cell. (C) Quantification of the percentage of attached bacteria to form an AE lesion. The standard deviation is indicated. *P* values were calculated with Student’s *t* test. *, *P* ≤ 0.05; **, *P* ≤ 0.005.

Because Cra, KdpE, and FusR affect LEE gene expression, which is necessary for AE lesion formation, we next assessed the roles of these transcription factors in the regulation of AE lesion formation. To form an AE lesion, EHEC uses specialized effectors to intimately attach to mammalian cells and reorganize actin to cup the bacteria, forming a pedestal-like structure. To assay whether deleting *cra*, *kdpE*, or *fusR* either alone or in combination affects EHEC AE lesion formation, HeLa cells were infected and assessed for the amount of AE lesions formed and the amount of bacteria attached to each lesion. Compared to WT bacteria (41.99 ± 5.46), the Δ*fusR* (56.74 ± 7.11) and Δ*fusR* Δ*kdpE* (61.52 ± 13.07) strains had significantly more bacteria attached to the HeLa cells, with 73.68, 70.67, and 77.44% of the attached bacteria able to form AE lesions, respectively (Fig. 1A to C). Any strain with *cra* deleted had significantly fewer bacteria attached to HeLa cells or associated with AE lesions. While the Δ*kdpE* strain had more attached bacteria (61.56 ± 16.74), with 69.86% forming pedestals compared to the WT, these data were not significant. Overall, these data indicate that Cra is a strong activator of AE lesion formation in EHEC; however, deleting *fusR* either alone or in combination with *kdpE* shows that these regulators repress or alter the kinetics of AE lesion formation prior to EHEC attachment to the HeLa cell.

### LEE gene expression under aerobic gluconeogenic conditions.

**FIG 2:**
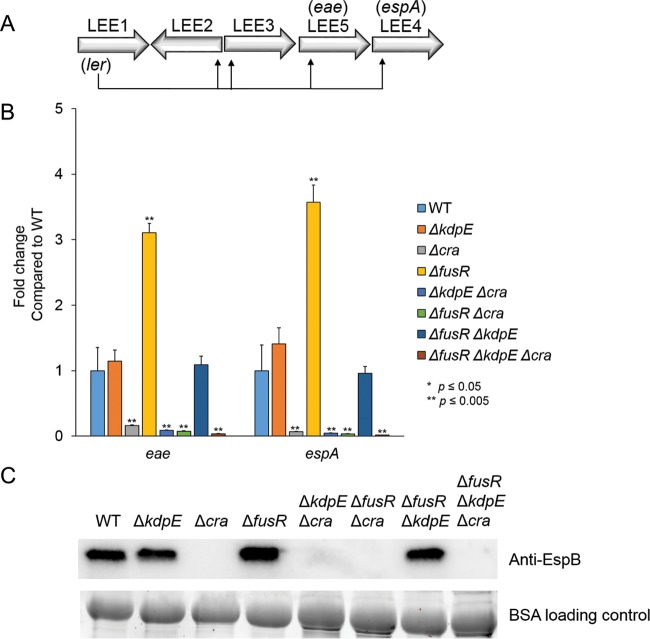
LEE gene expression in deletion strains under gluconeogenic, aerobic conditions with 1 mM pyruvate. (A) Schematic of the LEE operons. (B) Expression of representative LEE genes from strains grown aerobically with low-glucose DMEM with 1 mM pyruvate. (C) Western blot assay of EspB secreted from strains grown aerobically in low-glucose DMEM with 1 mM pyruvate. Significance was assessed with Student’s *t* test. BSA, bovine serum albumin.

AE lesion formation is dependent on LEE gene expression, and previous studies under aerobic gluconeogenic growth conditions showed that Cra and KdpE activate (34) while FusR represses LEE gene transcription (33). However, the potential cross talk among Cra, KdpE, and FusR has not been previously investigated. To assay whether deleting *cra*, *kdpE*, or *fusR* either alone or in combination affects LEE gene transcription, mutants were grown aerobically with low glucose and pyruvate, the same medium used for the assays described above. The expression of genes representative of two LEE operons, *eae* and *espA*, was measured. The Δ*cra*, Δ*kdpE* Δ*cra*, Δ*fusR* Δ*cra*, and Δ*fusR* Δ*kdpE* Δ*cra* strains had significantly reduced LEE gene expression (Fig. 2B). The Δ*kdpE* and Δ*fusR* Δ*kdpE* mutant strains had LEE gene expression levels slightly higher than but similar to those of the WT, while the Δ*fusR* strain had significantly enhanced LEE gene expression. To confirm our quantitative reverse transcription (qRT)-PCR data, Western blot assays were used to measure the secretion of the LEE-encoded effector EspB, which is encoded within the *LEE4* operon with *espA* (Fig. 2C). Taken together, these data again indicate that Cra strongly activates expression of the LEE while FusR represses it under these growth conditions. Of the three regulators, Cra has the most dominant effect on LEE gene expression.

**FIG 3:**
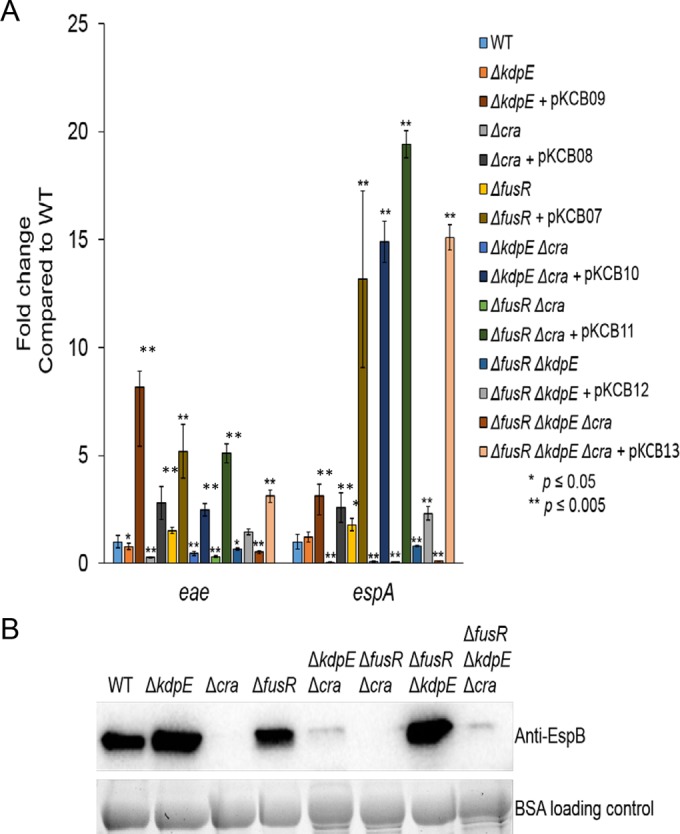
LEE gene expression in deletion strains under gluconeogenic, aerobic conditions. (A) Expression of representative LEE genes from strains and their respective complements grown aerobically in low-glucose DMEM. (B) Western blot assay of EspB secreted from strains grown aerobically in low-glucose DMEM. Significance was assessed with Student’s *t* test. BSA, bovine serum albumin.

As pyruvate is a central key metabolite and Cra activity is dependent on sensing of gluconeogenic conditions, the single, double, and triple mutants were grown aerobically with low glucose only to remove pyruvate as a potentially confounding variable. Independently of pyruvate, Cra still significantly reduced the expression of *eae* and *espA* in the Δ*cra*, Δ*kdpE* Δ*cra*, Δ*fusR* Δ*cra*, and Δ*fusR* Δ*kdpE* Δ*cra* strains (Fig. 3A). The expression of *eae* was also significantly decreased in the Δ*kdpE* strain, while *espA* expression was unchanged (Fig. 3A). These results are consistent with our previous report (34). EspA is encoded within the *LEE4* operon, whose expression is subject to high levels of posttranscriptional regulation, including the RNA binding protein CsrA (42–45). Consequently, a potential explanation for the differential regulation of *espA* (*LEE4*) expression in the *kdpE* mutant in regard to *eae* (Fig. 3A) and *ler* (34) is probably posttranscriptional regulation of the *LEE4* operon. In addition, secretion of EspB (which is cotranscribed with *espA* within the same operon) is significantly reduced in Δ*cra*, Δ*kdpE* Δ*cra*, Δ*fusR* Δ*cra*, and Δ*fusR* Δ*kdpE* Δ*cra* (Fig. 3B), indicating that Cra is an activator of LEE gene expression independent of pyruvate. Surprisingly, deleting *fusR* produced *eae* gene expression levels similar to WT, while *espA* levels were significantly elevated in the *fusR* mutant, suggesting that pyruvate somehow affects how FusR represses the LEE. The corresponding Western blot assays for secreted EspB showed that the Δ*fusR* strain had levels similar to those of the WT, while the Δ*kdpE* and Δ*fusR* Δ*kdpE* strain levels were slightly higher than those of the WT (Fig. 3B). These data suggest that pyruvate affects the degree of FusR repression of the LEE when oxygen is present. Potentially, EHEC could experience such conditions once the gut epithelium is disrupted by cell death, causing an infusion of blood, rich in both oxygen and pyruvate, to enter the environment. However, at some point in the infection, EHEC may need to repress its T3SS to move on to its next niche environment and FusR may help with such a transition.

To affirm that the deletion strains were not exhibiting polar effects, all strains were complemented and grown under aerobic gluconeogenic conditions. The expression of *eae* and *espA* by the complemented strains was measured (Fig. 3A).

### Effects of pyruvate on LEE gene transcription and secretion of EspB.

**FIG 4:**
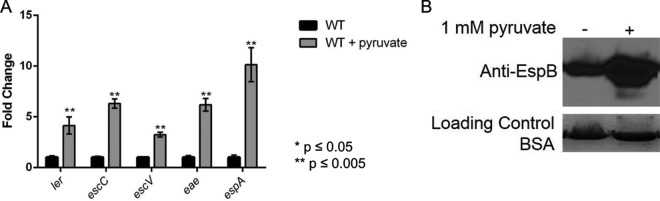
LEE gene expression in WT EHEC under gluconeogenic, aerobic conditions. (A) Expression of representative LEE genes from WT EHEC grown aerobically in low-glucose DMEM with or without 1 mM pyruvate. (B) Western blot assay of EspB secreted from strains grown aerobically in low-glucose DMEM with or without 1 mM pyruvate. Significance was assessed with Student’s *t* test.

Given the effect of pyruvate on FusR and its regulation of LEE gene expression, we investigated further how pyruvate may affect WT EHEC. To test if pyruvate also affects LEE gene expression, WT EHEC was grown aerobically in low-glucose DMEM with or without 1 mM pyruvate. Addition of pyruvate resulted in overexpression of all of the LEE genes tested and increased the secretion of EspB (Fig. 4A and B). This difference in expression is not due to a growth advantage, as EHEC has similar generation times when grown with or without pyruvate (see Fig. S2 in the supplemental material). While these conditions do not accurately mimic the conditions found in flowing human blood, the results may indicate that once EHEC infections become bloody in the intestines, the influx of oxygen and pyruvate may affect how EHEC regulates LEE gene expression. This is likely a dynamic process, with Cra, KdpE, and FusR shifting whether LEE gene expression is activated or repressed.

### Oxygen availability affects LEE gene transcription and secretion of EspB.

**FIG 5:**
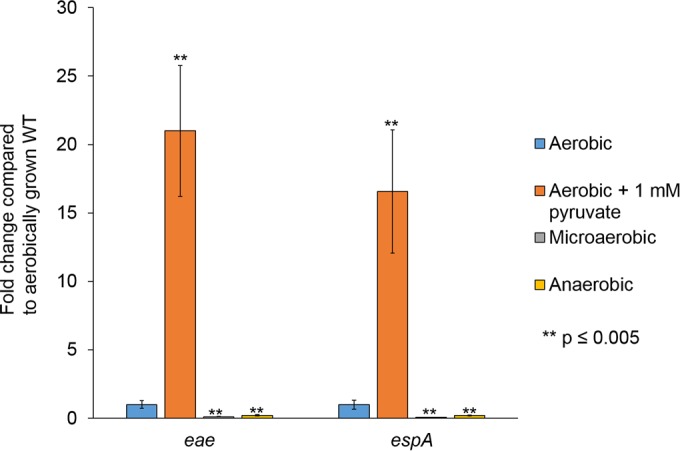
LEE gene expression in WT EHEC under different oxygen tensions. Expression of representative LEE genes by WT EHEC grown anaerobically, microaerobically, or aerobically in low-glucose DMEM with or without 1 mM pyruvate. Significance was assessed with Student’s *t* test.

In addition to the roles of pyruvate and sugar sources in LEE gene expression, oxygen availability in the gut varies from luminal to the epithelial surface (46, 47). We next asked if LEE gene transcription would be altered under anaerobic or microaerobic conditions. The intestinal lumen, the first site encountered by EHEC, is predominantly anaerobic (46, 47). As fewer bacteria are present near the epithelial apical surfaces, oxygen can diffuse across the enterocytes, creating a microaerobic environment (47). Recently, it was shown that the microbiota can also affect oxygen availability in the gut (48, 49). Moreover, *Citrobacter rodentium* (extensively used as a surrogate EHEC model for murine infections [50]), triggers colonic hyperplasia through the activity of the LEE-encoded T3SS, increasing oxygenation at the mucosal surface, leading to the aerobic expansion of *C. rodentium* (6). EHEC and *C. rodentium* intimately attach to enterocytes in an environment that may change from microaerobic to aerobic. The expression of the LEE *eae* and *espA* genes is lower under anaerobic conditions (growth in an anaerobic chamber), while it is enhanced under aerobic conditions and reaches even higher levels under aerobic conditions in the presence of pyruvate (Fig. 5).

**FIG 6:**
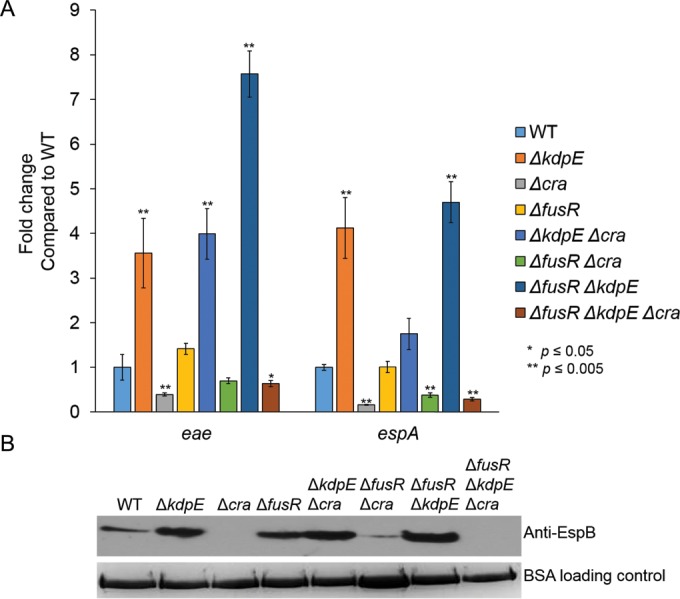
LEE gene expression in deletion strains under gluconeogenic, anaerobic conditions. (A) Expression of representative LEE genes from strains grown anaerobically in low-glucose DMEM. (B) Western blot assay of EspB secreted from strains grown anaerobically in low-glucose DMEM. Significance was assessed with Student’s *t* test. BSA, bovine serum albumin.

To investigate the contribution of Cra, FusR, and KdpE regulation under anaerobic conditions, we grew WT EHEC and the single, double, and triple mutants under anaerobic low-glucose conditions to measure the expression of the LEE genes, as well as the secretion of EspB. Unlike the aerobic conditions, KdpE had a strong repressive effect on *eae* and *espA* in the Δ*kdpE* and Δ*fusR* Δ*kdpE* strains, and in the Δ*kdpE* Δ*cra* strain, *eae* expression was significantly increased (Fig. 6A). Secretion of EspB was also enhanced in the Δ*kdpE*, Δ*kdpE* Δ*cra*, and Δ*fusR* Δ*kdpE* strains compared to that in the WT (Fig. 6B). FusR had expression levels similar to those of the WT (Fig. 6A). Cra again significantly reduced the expression of *eae* in the Δ*cra*, Δ*fusR* Δ*cra*, and Δ*fusR* Δ*kdpE* Δ*cra* strains, and the expression of *espA* in the Δ*cra* and Δ*fusR* Δ*kdpE* Δ*cra* strains (Fig. 6A). EspB secretion was significantly reduced in the Δ*cra*, Δ*fusR* Δ*cra*, and Δ*fusR* Δ*kdpE* Δ*cra* strains, again suggesting that Cra is a strong LEE activator (Fig. 6B).

**FIG 7:**
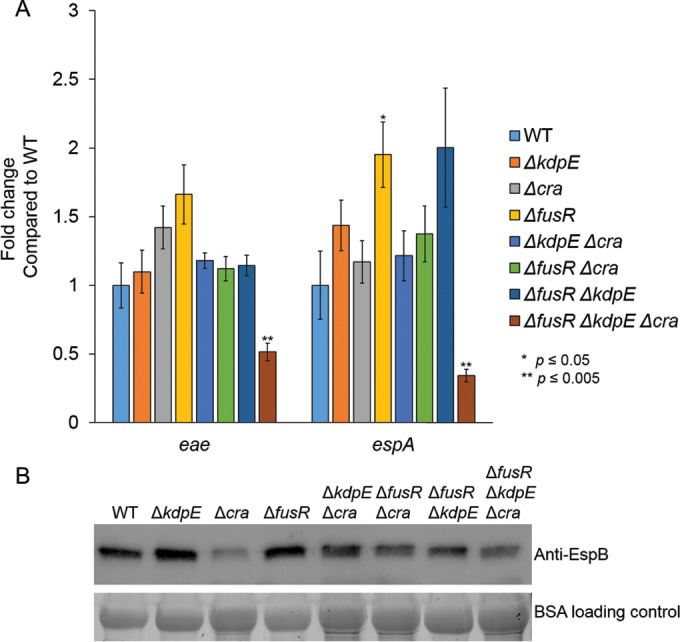
LEE gene expression in deletion strains under gluconeogenic, microaerobic conditions. (A) Expression of representative LEE genes by strains grown microaerobically in low-glucose DMEM. (B) Western blot assay of EspB secreted from strains grown microaerobically in low-glucose DMEM. Significance was assessed with Student’s *t* test. BSA, bovine serum albumin.

When we grew WT EHEC and all of the mutants under microaerobic conditions (the oxygen concentration was measured under microaerobic and aerobic conditions [see Fig. S5 in the supplemental material]), we saw the strong repressor and activator phenotypes become ameliorated, except in the Δ*fusR* Δ*kdpE* Δ*cra* strain (Fig. 7A). Secretion of EspB was not significantly different from that of the WT either (Fig. 7B). Overall, these data indicate that the presence or lack of oxygen significantly alters how EHEC regulates LEE gene expression through Cra, KdpE, and FusR.

### EHEC growth with different sugars found in the gut.

Because the gut has different oxygen concentrations, especially near the epithelial cell surface, and EHEC alters the expression of its virulence armamentarium dependent on the available oxygen, we next asked how limited oxygen availability affects the growth of EHEC on sugars available in the gut. EHEC and commensal *E. coli* predominantly consume monosaccharides (51–53). The mucin-derived sugars available for *E. coli* to utilize in the human gut include glucose, fucose, galactose, sialic acid, *N*-acetylgalactosamine, *N*-acetylglucosamine, fructose, xylose, and mannose (12, 54). However, the preference for which monosaccharides to metabolize differs between EHEC and commensal *E. coli* (52). EHEC strains defective in the following metabolism pathways have colonization defects compared to WT strains: fucose (Δ*fucAO*), ribose (Δ*rbsK*), mannose (Δ*manA*), arabinose (Δ*araBAD*), *N*-acetylglucosamine (Δ*nagE*), and galactose (Δ*galK*) (52). The ability to switch to alternative, less abundant carbon sources is therefore key for EHEC to colonize the gut.

Monosaccharides that feed into the Embden-Meyerhof-Parnas (EMP) pathway are a preferred carbon source for *E. coli*. However, pathogens often exploit nonpreferred carbon sources to gain a niche advantage. To measure the effects of different mucin-derived sugars from the large intestine, we grew WT EHEC and each of our deletion strains with individual sugars in DMEM and measured the overall generation times under oxygen-limited microaerobic conditions, an environmental condition most likely experienced as EHEC moves closer to the epithelial surface.

Under glycolytic (high-glucose) conditions, EHEC had a generation time of 78.2 ± 4.3 min (Table 1). Surprisingly, WT EHEC grown with galacturonic acid, an Entner-Doudoroff (ED) pathway sugar, had a generation time of 45.6 ± 0.7 min. The Δ*kdpE*, Δ*cra*, Δ*fusR*, and Δ*fusR* Δ*kdpE* mutant strains grown with galacturonic acid also had generation times that were shorter than those obtained with glucose. EHEC grew more slowly in all of the other sugars than in glucose. The majority of the mucin-derived sugars in which EHEC had generation times within 1 h longer than in glucose include xylose (103.2 ± 18.4), gluconic acid (108.9 ± 18.1 min), sialic acid (109.7 ± 8.9 min), *N*-acetylglucosamine (113.7 ± 12.7 min), mannose (115.6 ± 5.5 min), ribose (116.7 ± 2.2), glucuronic acid (120.9 ± 23.9), fucose (125.6 ± 48.1), galactose (134.9 ± 23.9), *N*-acetylgalactosamine (136.4 ± 23.0 min), and pyruvate (138.2 ± 12.7 min). Sugars in which EHEC had generation times within 2 h of those obtained with glucose include rhamnose (137.3 ± 4.5), arabinose (160.6 ± 13.4 min), and fructose (168.9 ± 24.9 min) (Table 1). All of the growth curves measured are shown in Fig. S3 and S4 in the supplemental material.

**TABLE 1:** Generation times of deletion strains in mucin-derived sugars

Sugar condition	Avg generation time (min) ± SD							
	WT	Δ*kdpE*	Δ*cra*	Δ*fusR*	Δ*kdpE* Δ*cra*	Δ*fusR* Δ*cra*	Δ*fusR* Δ*kdpE*	Δ*fusR* Δ*kdpE* Δ*cra*
0.1% glucose	90.5 ± 16.0	101.2 ± 12.6	85.9 ± 15.3	106.3 ± 14.0	67.0 ± 0.1	90.2 ± 9.1	78.2 ± 12.5	90.5 ± 2.2
0.4% glucose	78.2 ± 4.3	116.8 ± 29.1	75.0 ± 12.7	120.1 ± 37.5	71.4 ± 1.2	132.3 ± 9.0^a^	74.2 ± 20.5	102.2 ± 9.63
0.4% galactose	134.9 ± 23.9	199.6 ± 39.6	92.0 ± 28.9	197.0 ± 7.0	219.2 ± 9.2	183.7 ± 6.2	152.7 ± 21.3	164.9 ± 6.4
0.4% fructose	168.9 ± 24.9	211.0 ± 12.0	148.6 ± 3.6	233.4 ± 6.9	150.5 ± 26.6	191.0 ± 12.7	160.7 ± 31.6	169.5 ± 5.5
0.4% mannose	115.6 ± 5.5	113.5 ± 3.1	104.3 ± 25.7	137.6 ± 18.0	82.4 ± 13.9	124.4 ± 26.8	108.0 ± 7.9	116.8 ± 19.3
0.4% *N*-acetylglucosamine	113.7 ± 12.7	174.2 ± 6.0	130.2 ± 8.8	184.4 ± 2.7	164.9 ± 7.1	146.9 ± 28.5	134.4 ± 10.2	139.0 ± 19.1
0.4% *N*-acetylgalactosamine	136.4 ± 23.0	170.3 ± 34.5	196.7 ± 66.1	153.3 ± 7.9	135.6 ± 40.7	175.6 ± 41.5	119.6 ± 11.3	253.2 ± 29.6
0.4% sialic acid	109.7 ± 8.9	131.8 ± 15.3	139.3 ± 7.9	148.1 ± 13.0	121.6 ± 7.7	144.0 ± 36.6	116.2 ± 38.9	145.1 ± 28.2
0.4% rhamnose	137.3 ± 4.5	174.1 ± 21.3	133.4 ± 21.4	151.6 ± 28.5	98.0 ± 10.7	103.2 ± 3.3^a^	106.3 ± 7.0^a^	121.5 ± 12.8
0.4% fucose	125.6 ± 48.1	186.2 ± 0.9	270.3 ± 142.2	138.7 ± 18.8	138.7 ± 18.8	167.5 ± 31.7	130.6 ± 20.0	204.6 ± 30.1
0.4% pyruvate	138.2 ± 12.7	172.3 ± 10.5	None	171.9 ± 9.3	None	None	109.0 ± 12.2	None
0.4% galacturonic acid	45.6 ± 0.7	59.9 ± 4.1	67.7 ± 7.6	52.1 ± 4.8	78.5 ± 11.4	103.4 ± 1.7^a^	52.8 ± 8.4	107.0 ± 3.7^a^
0.4% gluconic acid	108.9 ± 18.1	187.3 ± 13.5^a^	142.8 ± 24.0	157.0 ± 25.0	103.1 ± 36.4	123.2 ± 9.0	119.0 ± 5.4	164.1 ± 7.8
0.4% glucuronic acid	120.9 ± 23.9	187.3 ± 13.5	132.5 ± 38.5	150.4 ± 46.2	103.7 ± 35.5	121.3 ± 11.6	119.0 ± 5.4	153.1 ± 15.8
0.4% arabinose	160.6 ± 13.4	217.4 ± 11.9^a^	126.4 ± 10.8	204.6 ± 4.5	167.7 ± 31.1	135.9 ± 28.5	154.0 ± 32.5	128.3 ± 9.1
0.4% xylose	103.2 ± 18.4	130.5 ± 16.6	87.6 ± 11.6	168.6 ± 25.7	108.9 ± 50.3	104.7 ± 31.0	120.6 ± 66.5	122.4 ± 29.3
0.4% ribose	116.7 ± 2.2	198.1 ± 1.6^a^	217.5 ± 7.3^a^	185.0 ± 6.0^a^	195.6 ± 16.0	258.0 ± 24.8	176.3 ± 15.7	96.6 ± 12.3

a Significantly different (*P* > 0.05, Student’s *t* test) from the WT grown with the sugar indicated.

None of the strains harboring mutations in *kdpE*, *cra*, or *fusR* grew significantly better than WT EHEC. We were surprised that the Δ*fusR* mutant did not grow faster than the WT when grown in fucose only, as was previously shown for aerobically grown cultures (33). This difference may result from growing this mutant under microaerobic conditions, further indicating the sensitivity of these transcription factors to the available oxygen. However, for six of the sugars, strains with mutations had generation times significantly worse than those of the WT grown with the same carbon source (Table 1). For pyruvate, it should be noted that strains harboring a Δ*cra* mutation failed to grow, as has been previously demonstrated (55).

### EHEC changes the secretion of a virulence protein when grown with different sugars.

**FIG 8:**
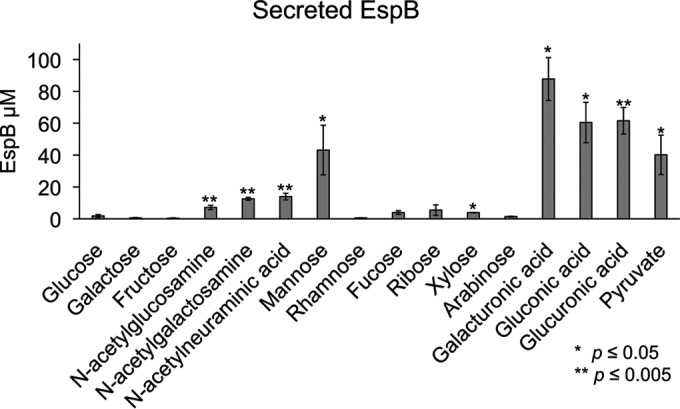
Secretion of EspB by bacteria grown under microaerobic conditions with 0.4% mucin-derived sugar as the sole carbon source. Significance was assessed with Student’s *t* test.

Given the differences in the generation times of WT EHEC with different mucin-derived sugars, we next asked whether these different sugars affect the expression of the LEE-encoded protein EspB. The WT EHEC and Δ*espB* mutant strains were grown to early stationary phase under microaerobic conditions before growth was halted. With all of the sugars tested, the Δ*espB* mutant strain grew similarly to WT EHEC. As expected, WT EHEC grown under high-glucose conditions expressed low concentrations of EspB (Fig. 8). Low EspB production was also observed when galactose, fructose, rhamnose, fucose, ribose, xylose, or arabinose was the sole carbon source. Secretion of EspB increased substantially when galacturonic acid, gluconic acid, glucuronic acid, pyruvate, sialic acid, or mannose was the sole carbon source. A modest increase in EspB secretion was observed when *N*-acetylglucosamine and *N*-acetylgalactosamine were used. These data demonstrate that the utilization of at least eight alternative sugars by EHEC can promote the expression and secretion of virulence genes.

## DISCUSSION

The GI tract is composed of varied environments and niches that are colonized by different microbial communities. These differences in the gut biogeography of the microbiota are largely dictated by differences in nutrient availability and oxygen tension (1). It is fundamentally important for enteric pathogens to inform themselves of their location and the available resources within the gut and translate this information to regulate the expression of their virulence repertoire.

EHEC has a very small infectious dose, estimated to be 50 CFU (14). Consequently, it is of paramount importance for EHEC to correctly determine its location and the resources available to regulate the expression of virulence genes. EHEC regulation of the LEE pathogenicity island is complex and involves the sensing of both microbiota- and host-derived signals and metabolites. The transcription factors Cra, KdpE, and FusR play an important role in the integration and sensing of these environmental cues, leading to optimal regulation of the LEE genes (7, 32, 35, 38, 56). EHEC colonizes the colon, where the major source of carbon is the mucus, which is decorated with sugars such as fucose. These sugars can be harvested by saccharolytic members of the microbiota, such as *Bacteroides thetaiotaomicron*, and made available to other bacterial species that lack this capability (10). *B. thetaiotaomicron* causes a remarkable modification of the metabolic landscape in the gut, leading to enhanced concentrations of succinate and metabolites that characterize a gluconeogenic signature (34). These are sensed by Cra, which acts in concert with KdpE to directly activate LEE gene expression (7, 34). There is also an additional interplay among Cra, KdpE, and FusR, whose cognate HK (FusK) senses fucose cleaved from the mucus by *B. thetaiotaomicron* (33). *B. thetaiotaomicron* is also important for liberating many other sugars from the mucosal glycoconjugates, including sialic acid, *N*-acetylneuraminic acid, and others (57). Here we examined how specific mucin-derived sugars liberated by the microbiota affect EHEC generation times and the secretion of virulence proteins. EHEC has the shortest generation time in the ED pathway sugar galacturonic acid, which was twice as short as the generation time in glucose. In addition, the most abundant secretion of EspB was observed with the tested ED pathway sugars (Fig. 8; see Fig. S3 and S4 in the supplemental material). Utilization of the ED pathway may help EHEC compete among anaerobes that rely on the EMP pathway (classical glycolysis) for ATP production (58).

ED pathway sugars are common in the large intestine from the metabolism of pectin, which is present in fruit and vegetable cell walls. Pectin is constructed of long chains of α-1,4-glycoside-linked d-galacturonic acid that are decorated with other terminal sugars such as rhamnose, d-xylose, l-fucose, d-glucuronic acid, and others (59). Importantly, pectin is degraded only in the large intestine, where *B. thetaiotaomicron* and other microbiota species encode the enzymes to break the long or branched side chains (60, 61). *E. coli* also degrades smaller pectin substrates when *B. thetaiotaomicron* is present in the large intestine (62). Thus, the amount of galacturonic acid can remain high in diets steady in plant fiber (61). The short generation time of EHEC when galacturonic acid is the sole carbon source and under limited-oxygen conditions and the increased secretion of LEE-encoded EspB suggest that the ED pathway may help initiate EHEC infection and that diet may affect the outcome of EHEC infections.

Oxygen availability dictates the utilization of different metabolic pathways and is important in enteric infections (6, 63–66). As EHEC moves through the large intestinal lumen, it encounters primarily anaerobic conditions (46, 47, 61, 64, 67). This radial oxygen gradient is dependent on atmospheric pressure, the host’s ability to sequester oxygen, and the aerotolerant members of the microbiota consuming oxygen in the outer mucosal layer (46, 48, 68). When EHEC moves toward the gut epithelial cells, oxygen concentrations increase because of diffusion across the microvillus capillary network, thus creating a microaerobic environment (46, 47, 66, 68, 69). Indeed, an *in vitro* model of polarized human epithelial cells indicates that the expression of LEE-encoded EspA, EspB, and Tir increases and promotes adherence under microaerobic conditions (66). Moreover, in murine infections that promote host inflammation, aerotolerant species such as *C. rodentium* and *Campylobacter jejuni* blossom when the host microbiota is eliminated or reduced, resulting in higher oxygen concentrations (70). Low butyrate concentrations, which would be associated with a disrupted microbiota, also promote the expression of the T3SS in EHEC (71). Recently, the expression of the LEE-encoded T3SS in *C. rodentium* (an EHEC surrogate murine infection model extensively employed in the field) was directly tied to its ability to promote an increase in host oxygen availability, which allowed the bacteria to bloom and exploit nutrient niches occupied by the microbiota (6). This increase in oxygen could explain the expansion of aerobic *Proteobacteria* during a *C. rodentium* infection (70), which ultimately, through competition for nutrient sources, limits the *C. rodentium* infection (51). Together, these data suggest that variations in the concentration of oxygen should be sensed by EHEC to successfully control virulence gene expression.

Oxygen availability affects LEE gene expression in WT EHEC and also how Cra, KdpE, and FusR alter expression of the LEE (Fig. 2 to 7). The LEE is poorly expressed under anaerobic conditions and optimally expressed under aerobic conditions, especially in the presence of pyruvate (Fig. 5). Under anaerobic conditions, KdpE and FusR are repressors of LEE gene expression, while Cra activates LEE gene expression (Fig. 6). EHEC encounters an anaerobic environment near the lumen or the outer mucosal layer—an unfavorable location at which to express a T3SS. Microaerobic conditions support LEE gene expression for all of our mutant strains (Fig. 7). These data suggest that the limited amount of oxygen available is most similar to moving toward or having close contact with intestinal epithelial cells (46, 47, 66). This site is where EHEC should optimally express its T3SS. Of note, oxygen availability is higher at this site and increases with the infection (6). When oxygen is readily available under aerobic conditions, Cra is a strong activator of the LEE (Fig. 2 and 3) and this activation may be enhanced by its physical interaction with KdpE (34). These conditions would mimic disruption of the intestinal epithelial barrier and infusion of blood into the gut. Surprisingly, FusR is more repressive when pyruvate is present. Pyruvate may signal that the expression of a T3SS is no longer warranted and perhaps EHEC should move on to another environmental niche.

In summary, oxygen availability and utilization of nonpreferred mucin-derived carbon sources affect the ability of EHEC to express its T3SS. EHEC is versatile in sensing a plethora of environmental cues to accurately time the deployment of the energetically costly T3SS. The savviness of EHEC in highjacking ancient metabolic systems to deploy its virulence armamentarium highlights the deftness of enteric pathogens in causing disease and their ability to precisely sense and adapt to microniches within the intestine.

## MATERIALS AND METHODS

### Strains and plasmids.

The strains and plasmids used in this study are listed in Table S1 in the supplemental material. Standard molecular biology methods were used (72). The primers used are listed in Table S2 in the supplemental material. Nonpolar mutants were constructed by using the λ Red protocol so that all would be in the same genetic background (73). To construct strains harboring a Δ*cra* mutation, a PCR product was amplified with NEB Phusion polymerase and primers JcraredF and JcraredR (see Table S2 in the supplemental material). For a Δ*kdpE* mutation, a PCR product was amplified with primers kdpEλRed-F and kdpEλRed-R (see Table S2 in the supplemental material). For a Δ*fusR* mutation, a PCR product was amplified with primers Z0463lambdaredP1 and Z0463lambdaredP2. All deletion PCR products used pKD4 as the template and were gel purified (Qiagen). The PCR product was then electroporated into the prepared cells and recovered in S.O.C. medium (2% tryptone, 0.5% yeast extract, 10 mM NaCl, 2.5 mM KCl, 10 mM MgCl_2_, 10 mM MgSO_4_, 20 mM glucose) for 3 h at 30°C and plated on LB containing kanamycin overnight at 37°C. Colonies were then screened for ampicillin sensitivity and kanamycin resistance and PCR verified with primers for *cra* (KCB topo cra FW/KCB topo cra RV), *kdpE* (KCB topo kdpE FW/KCB topo kdpE RV), or *fusR* (Z0463for/Z0463rev) for the absence of the gene (see Table S2 in the supplemental material). To create nonpolar mutants, the kanamycin resistance cassette was resolved with resolvase plasmid pCP20. The mutants were electroporated with pCP20, and resultant colonies were patched for kanamycin sensitivity. Final verification of proper deletion was performed by sequencing.

To construct the complementation plasmids, each gene was cloned individually into the Invitrogen pCR-Blunt II-TOPO plasmid in accordance with the manufacturer’s instructions. PCR products to clone into the TOPO plasmid were amplified with NEB Phusion polymerase with EHEC 86-24 as the template. The primers used to amplify the PCR products of *cra* (KCB topo cra FW/KCB topo cra RV), *kdpE* (KCB topo kdpE FW/KCB topo kdpE RV), and *fusR* (Z0463for/Z0463rev) are described in Table S2 in the supplemental material. To construct complemented strains that express each gene equally, NEBuilder cloning was used to place each gene under the control of the P_cat_ promoter. Briefly, puc19 was linearized with NEB restriction enzyme Eco53kI. With the appropriate TOPO vector (KCB01, KCB02, or KCB03) as a template, each gene was PCR amplified with NEB Phusion polymerase as follows (from Table S2 in the supplemental material): *cra*, Pcat to cra FW/Puc19 to cra RV; *kdpE*, pcat to kdpE FW/puc19 to kdpE RV; *fusR*, pcat to fusR FW/puc19 to fusR RV. With pACYC184 as the template, the P_cat_ promoter for each gene was amplified as follows (from Table S2 in the supplemental material): *cra*, puc19 to pcat FW/cra to pcat RV; *kdpE*, puc19 to pcat FW/kdpE to pcat RV; *fusR*, puc19 to pcat FW/fusR to pcat RV. Linearized puc19 and the PCR products for each gene and the corresponding P_cat_ promoter PCR products were gel purified (Qiagen) prior to incubation at 50°C for 20 min with the NEBuilder HiFi DNA Assembly master mix in accordance with the manufacturer’s instructions. Ligated products were then diluted and electroporated into DH5α cells. Cells recovered at 37°C for 1 h. Transformants were selected for ampicillin resistance and confirmed by sequencing with M13 RV as a primer. These plasmids are pKCB04, pKCB05, and pKCB06.

To construct the complement vectors for each single gene in pACYC184, the plasmid was digested with NEB enzymes EcoRV and SalI. pKCB04 was digested with SfoI and SmaI, pKCB05 was digested with SfoI and SalI, and pKCB06 was digested with SfoI and SalI (NEB). The digested template and respective genes were gel purified (Qiagen) before ligation with NEB T4 ligase overnight at 16°C. Ligation products were electroporated into DH5α cells that were allowed to recover at 37°C for 1 h. Transformants were selected for chloramphenicol resistance and tetracycline sensitivity prior to confirmation by sequencing with the appropriate primers used as described above. These plasmids were then transformed into the corresponding single-deletion EHEC strains to construct KCB07, KCB08, and KCB09.

To construct the remaining complement vectors in low-copy-number plasmid pACYC184, the NEBuilder method was again used to make the double- and triple-gene complements. pACYC184 was linearized with NEB EcoRV and gel purified (Qiagen). To make the *kdpE cra* complement (pKCB10), PCR amplification of the pKCB05 template was done with primers p184kdpE to cra FW and p184 to cra RV and pKCB06 was PCR amplified with primers p184 to kdpE FW and p184 cra to kdpE RV. To make the *fusR cra* complement (pKCB11), PCR amplification of the pKCB04 template was done with primers p184 to fusR FW and p184cra to fusR RV and the pKCB05 template was PCR amplified with primers p184fusR to cra FW and p184 to cra RV. To make the *fusR kdpE* complement (pKCB12), PCR amplification of the pKCB06 template was done with primers p184 to kdpE FW and p184fusR to kdpE RV and the pKCB04 template was PCR amplified with primers p184kdpE to fusR FW and p184 to fusR RV. To make the *fusR kdpE cra* complement (pKCB13), PCR amplification of the pKCB06 template was done with primers p184 to kdpE FW and p184fusR to kdpE RV, the pKCB04 template was PCR amplified with primers p184kdpE to fusR FW and p184cra to fusR RV, and the pKCB05 template was PCR amplified with primers p184fusR to cra FW and p184 to cra RV. Each PCR product was gel purified (Qiagen) prior to incubation with linearized pACYC184 and NEBuilder HiFi DNA Assembly master mix at 50°C for 1 h. Ligated products were then diluted and electroporated into DH5α cells. Cells recovered at 37°C for 1 h. Transformants were selected for chloramphenicol resistance and tetracycline sensitivity prior to confirmation by sequencing with the appropriate primers used as described above. These plasmids were then transformed into the corresponding EHEC deletion strains to construct KCB09, KCB10, KCB11, and KCB12.

### RNA extraction and qRT-PCR.

For EHEC strains grown aerobically and shaking, cultures were grown in 0.1% glucose with or without 1 mM pyruvate DMEM to an optical density at 600 nm (OD_600_) of 1.0. Complemented EHEC strains were grown aerobically with shaking in 0.1% glucose DMEM to an OD_600_ of 1.0. For EHEC strains grown statically (microaerobic) or in an anaerobic chamber, cultures were grown for 6 h to early stationary phase in 0.1% glucose DMEM to an OD_600_ of 0.6. RNA from three replicates was extracted with the RiboPure bacterial isolation kit in accordance with the manufacturer’s protocols (Ambion). qRT-PCR was performed as described previously (38). Briefly, diluted extracted RNA was mixed with validated primers (see Table S2 in the supplemental material), RNase inhibitor, and reverse transcriptase (AB). The mixture was used in a one-step reaction utilizing an ABI 7500 sequence detection system. Data were collected with ABI Sequence detection 1.2 software, normalized to endogenous *rpoA* levels, and analyzed by the comparative critical threshold (*C*_*T*_) method. Analyzed data are presented as fold changes over WT levels. The Student unpaired *t* test was used to determine statistical significance. A *P* value of <0.05 was considered significant.

### Western blot assays for secreted proteins.

From cultures grown in 0.1% glucose with or without 1 mM pyruvate DMEM, secreted proteins were isolated as previously described (18). Twenty micrograms of bovine serum albumin was added to secreted protein samples as a loading control. Proteins were separated by 12% SDS-PAGE, transferred to a polyvinylidene fluoride membrane, and blocked with 10% milk in phosphate-buffered saline (PBS) containing 0.05% Tween (PBST). Membranes were probed with an anti-EspB primary antibody, washed, and then incubated with a secondary antibody conjugated to streptavidin-horseradish peroxidase. GE enhanced-chemiluminescence reagent was added, and the membranes were exposed either to film or with the Bio-Rad ChemiDoc Touch Imaging System (software 1.0.0.15) with Image Lab 5.2.1 software for image display. Each growth condition was replicated a minimum of three times.

### Fluorescein actin staining assays.

Fluorescein actin staining assays were performed as previously described (74). Briefly, HeLa cells were grown overnight to about 80% confluence at 37°C in 5% CO_2_ on coverslips in wells containing DMEM supplemented with 10% fetal bovine serum and a 1% penicillin-streptomycin-glutamine antibiotic mixture. Prior to infection, fresh medium lacking antibiotics replaced the overnight medium. To infect HeLa cells, overnight static bacterial cultures were diluted 100:1 (bacteria to DMEM). After 3 h of infection, the wells were again replaced with fresh medium lacking antibiotics. After 6 h of infection, the coverslips were washed, fixed, and permeabilized. The samples were treated with fluorescein isothiocyanate-labeled phalloidin to visualize actin accumulation and propidium iodide to visualize bacterial DNA and HeLa cell nuclei, respectively. The coverslips were then mounted on slides and imaged with a Zeiss Axiovert microscope. Pedestal formation was quantified as the percentage of pedestals formed per attached bacterium. Replicate coverslips from multiple experiments were quantified, and statistical analyses were performed with Student’s unpaired *t* test. Serially diluted samples of the original bacterial cultures were also plated to confirm that similar CFU ratios were used for infection.

### Microaerobic growth curves and generation times with mucin-derived sugars.

Overnight cultures were diluted 1:100 into 96-well Costar plates in a 0.4% concentration of the sugar indicated in DMEM. The sugars, all purchased from Sigma, included d-(+)-xylose, sodium pyruvate, d-(–)-fructose, l-rhamnose monohydrate, d-(–)-ribose, l-(–)-fucose, d-(+)-galactose, *N*-acetyl-d-galactosamine, *N*-acetyl-d-glucosamine, *N*-acetylneuraminic acid, d-glucuronic acid sodium salt monohydrate, d-galacturonic acid sodium salt, d-gluconic acid sodium salt, d-(+)-mannose, d-(+)-glucose, and l-(+)-arabinose. The OD_600_ was measured every hour for the first 8 h and again at 24 h of growth with a BMG Labtech FLUOstar Optima microplate reader (software version 2.10 R2, firmware version 1.24). Data were plotted both linearly and semilogarithmically. Generation times (*Gt*) were calculated from the linear portion of the semilogarithmic curves as follows: *Gt* = time × log(2)/(log_final concentration_ − log_initial concentration_).

### Protein purification and ELISA.

pET21-based plasmids expressing EspB were induced with isopropyl-β-d-thiogalactopyranoside (IPTG), and the proteins were purified with nickel columns (Qiagen). His-EspB was buffered exchanged to PBS with Amicon filters before being diluted for standard control. For the enzyme-linked immunosorbent assay (ELISA), overnight WT and Δ*espB* mutant EHEC cultures were diluted 1:100 into 96-well plates in a 0.4% concentration of the sugar indicated in DMEM. The OD_600_ was measured at early stationary growth phase with a BMG Labtech FLUOstar Optima microplate reader to confirm that the two strains were comparable in growth. Bacterial growth was quenched with 4× STOP solution (0.92 M sodium azide and 100 μl of Sigma protease cocktail inhibitor in PBS). Quenched reaction mixtures were diluted 1:2 and incubated in Dynatech Laboratories Microtiter ELISA plates. Wells were blocked with 5% milk in PBST. Samples were washed in PBST prior to incubation with an anti-EspB primary antibody and a secondary antibody conjugated to streptavidin-horseradish peroxidase. Plates were developed with Sigma 3,3′,5,5′-tetramethylbenzidine and stopped with 2N HCl. The OD_450_ was measured with a BMG Labtech FLUOstar Optima microplate reader, and EspB concentrations were calculated on the basis of known control protein standard curves. The Student unpaired *t* test was used to determine statistical significance. A *P* value of <0.05 was considered significant.

## SUPPLEMENTAL MATERIAL

Figure S1  Growth curves of WT and deletion strains. (A) Strains were grown in LB with shaking at 250 rpm at 37°C. No significant differences between the growth rates of the WT and deletion strains were measured. (B) Strains were grown under gluconeogenic conditions with shaking at 250 rpm at 37°C in DMEM with 1 g/liter glucose and 1 mM pyruvate. Download Figure S1, PDF file, 0.3 MB

Figure S2  Growth curves of WT and deletion strains. (A) Strains were grown microaerobically under gluconeogenic conditions in DMEM with 1 g/liter glucose with or without 1 mM pyruvate in microtiter plates at 37°C. No significant differences between the growth rates of the WT and deletion strains were measured. Download Figure S2, EPS file, 1.5 MB

Figure S3  Growth curves of WT and deletion strains grown microaerobically with 0.4% EMP pathway sugar in DMEM in microtiter plates at 37°C. Download Figure S3, PDF file, 0.3 MB

Figure S4  Growth curves of WT and deletion strains grown microaerobically with 0.4% pentose phosphate pathway or ED pathway sugar in DMEM in microtiter plates at 37°C. Download Figure S4, EPS file, 2.5 MB

Figure S5  Oxygen concentrations for WT EHEC grown microaerobically or anaerobically in 0.1% glucose (A), 0.1% glucose with 1 mM pyruvate (B), or 0.1% pyruvate DMEM (C) at 37°C. Oxygen measurements were made with a Unisense oxygen microsensor and multimeter version 2.01 in accordance with the manufacturer’s instructions. Download Figure S5, PDF file, 0.1 MB

Table S1  Strains and plasmids used in this study. Table S1, DOCX file, 0.01 MB

Table S2  Oligonucleotides and qRT-PCR primers used in this study. Table S2, DOCX file, 0.01 MB
