# Supplementary figures and images for: Catabolite and Oxygen Regulation of Enterohemorrhagic Escherichia coli Virulence
Source: mBio. 2016 Nov 22;7(6):e01852-16. doi: 10.1128/mBio.01852-16 (PMC5120142; doi:10.1128/mBio.01852-16)

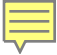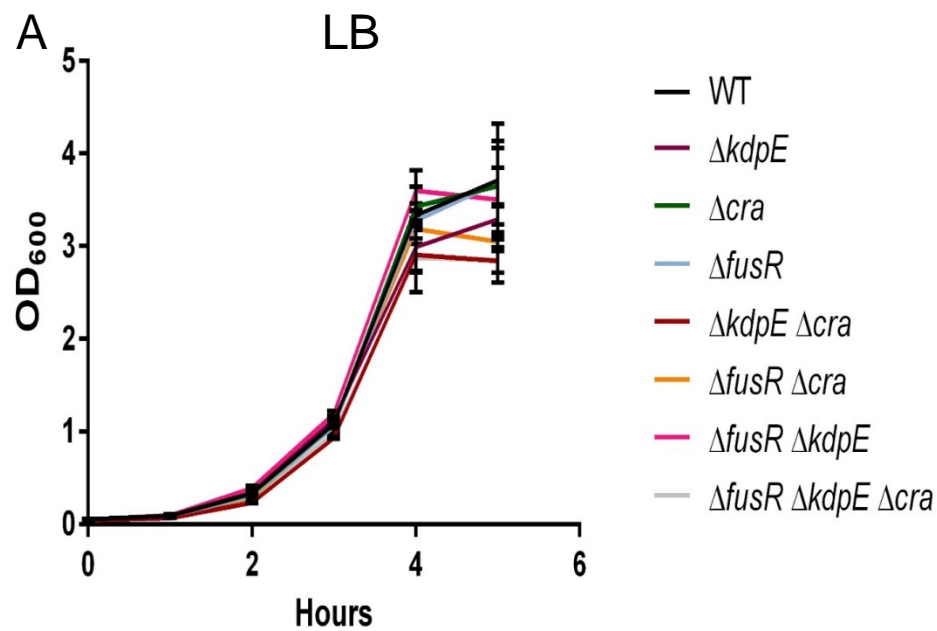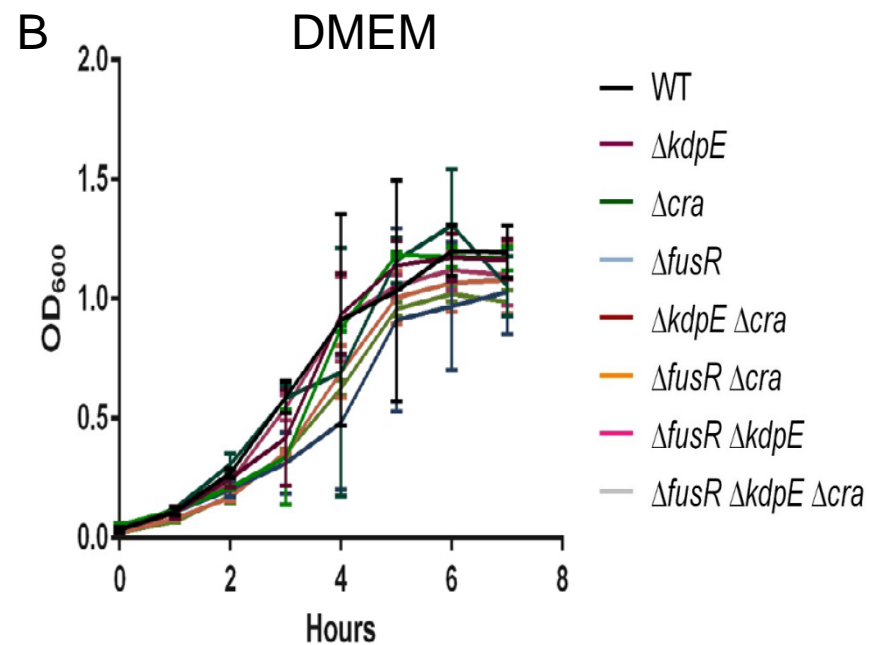

Supplement: Figure S1 — Growth curves of WT and deletion strains. (A) Strains were grown in LB with shaking at 250 rpm at 37°C. No significant differences between the growth rates of the WT and deletion strains were measured. (B) Strains were grown under gluconeogenic conditions with shaking at 250 rpm at 37°C in DMEM with 1 g/liter glucose and 1 mM pyruvate. Download [file mbo006163086sf1.pdf]

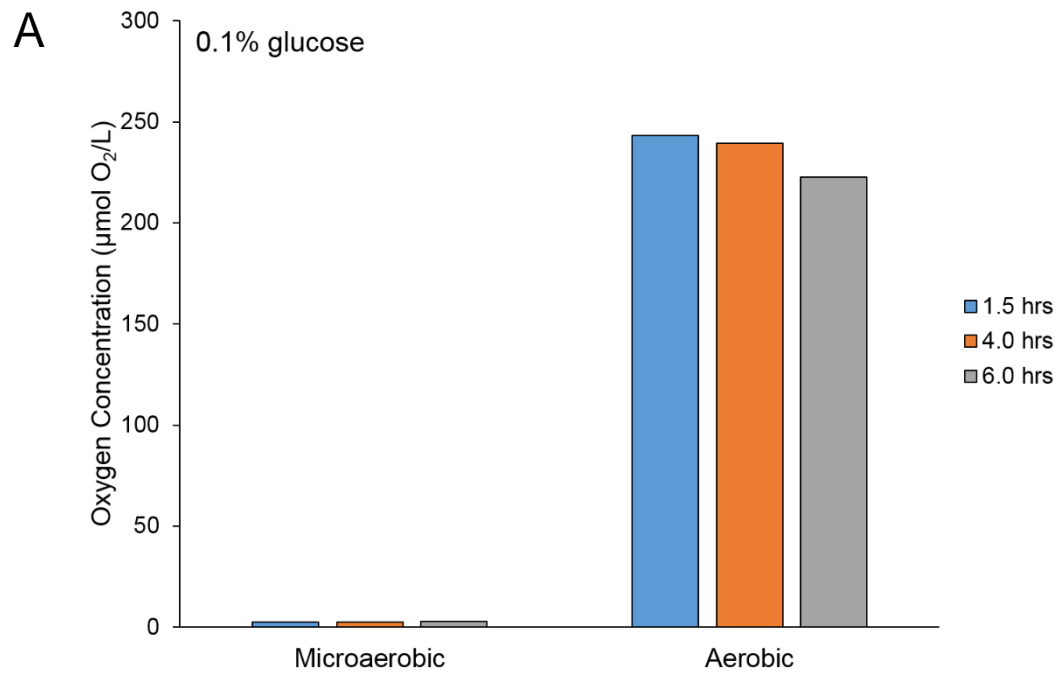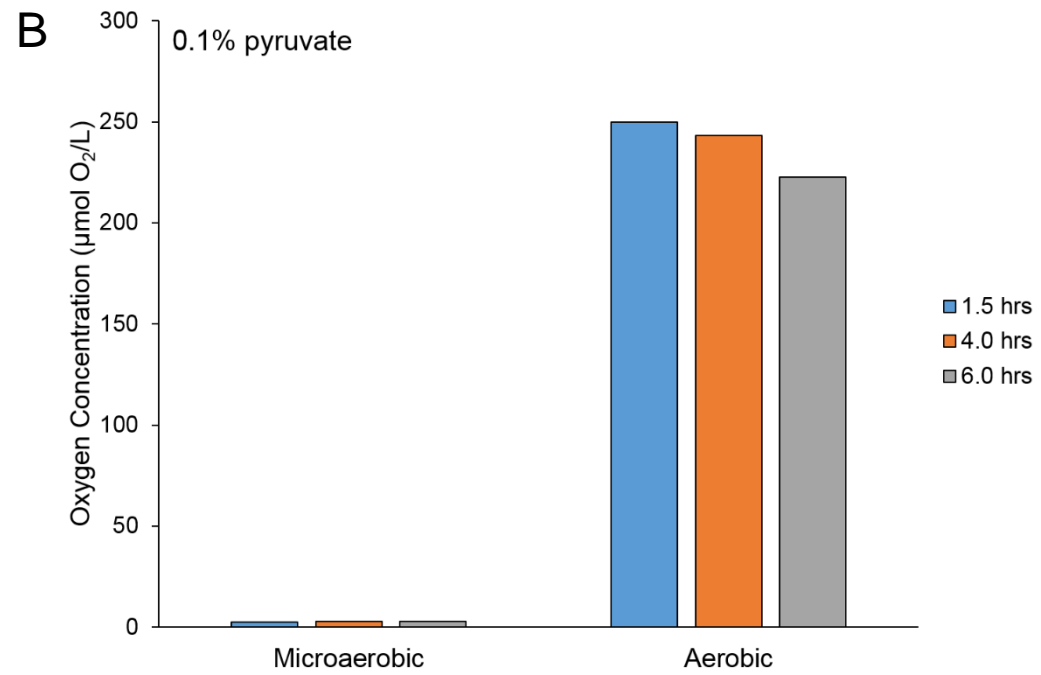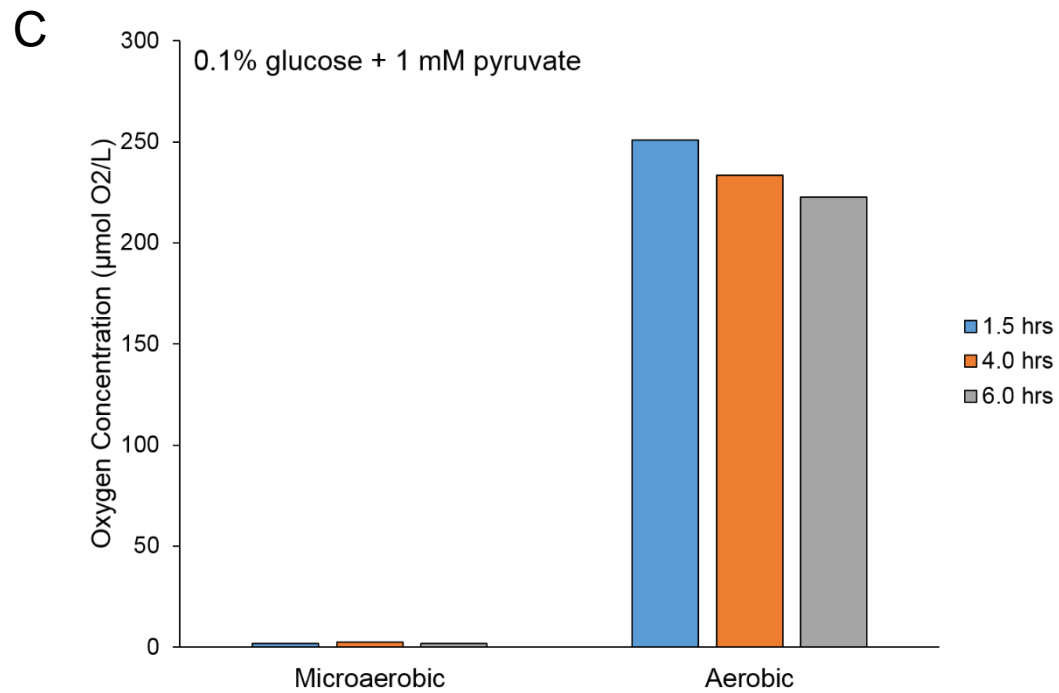

Supplement: Figure S5 — Oxygen concentrations for WT EHEC grown microaerobically or anaerobically in 0.1% glucose (A), 0.1% glucose with 1 mM pyruvate (B), or 0.1% pyruvate DMEM (C) at 37°C. Oxygen measurements were made with a Unisense oxygen microsensor and multimeter version 2.01 in accordance with the manufacturer’s instructions. Download [file mbo006163086sf5.pdf]
