# Supplementary material for: Catabolite and Oxygen Regulation of Enterohemorrhagic Escherichia coli Virulence
Source: mBio. 2016 Nov 22;7(6):e01852-16. doi: 10.1128/mBio.01852-16 (PMC5120142; doi:10.1128/mBio.01852-16)
Supplement: Figure S3 — Growth curves of WT and deletion strains grown microaerobically with 0.4% EMP pathway sugar in DMEM in microtiter plates at 37°C. Download [file mbo006163086sf3.pdf]

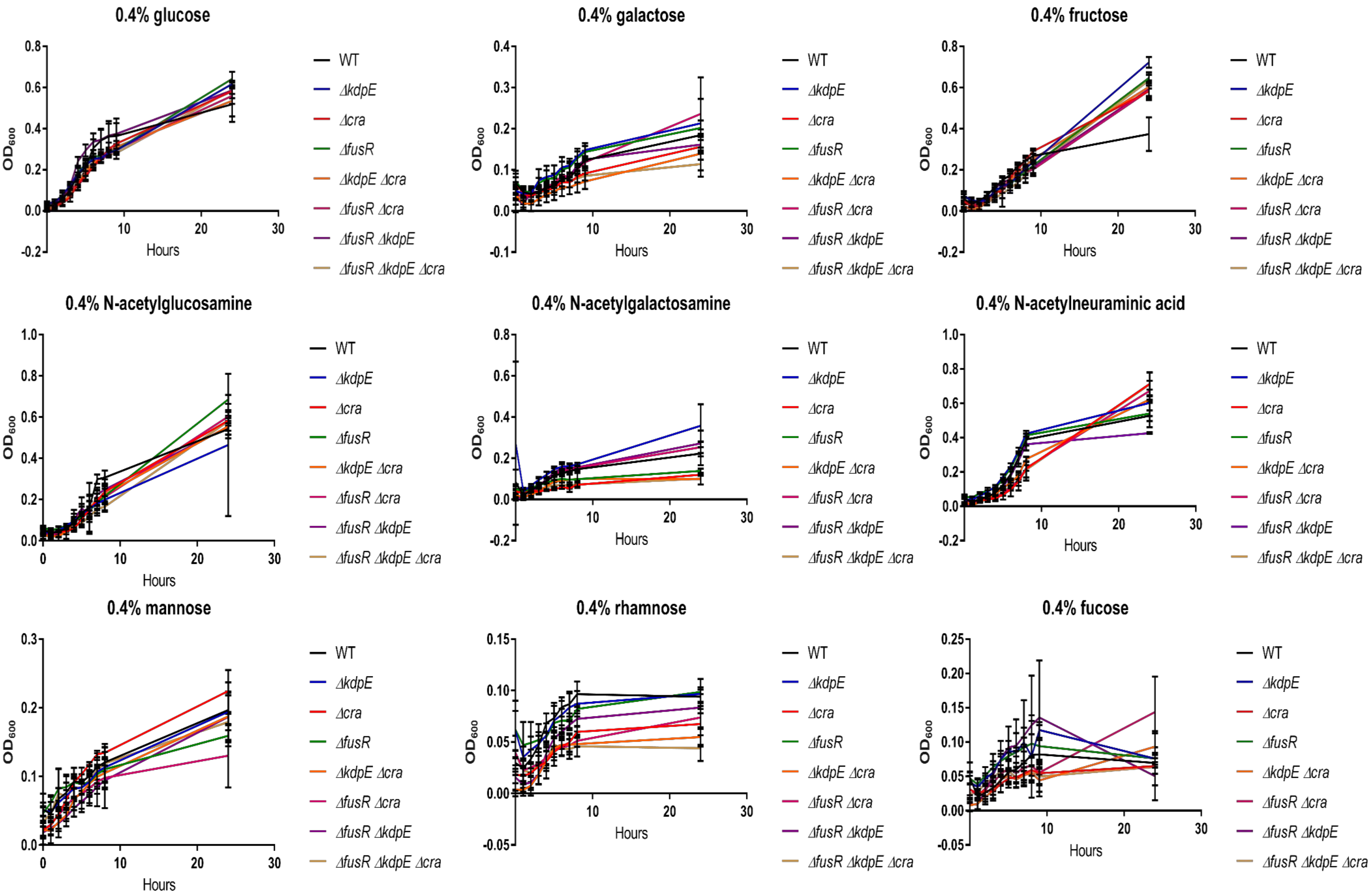
0.4% glucose
0.4% galactose
0.4% fructose
0.4% N-acetylglucosamine
0.4% N-acetylgalactosamine
0.4% N-acetylneuraminic acid
0.4% mannose
0.4% rhamnose
0.4% fucose
OD_{600}
Hours
WT
ΔkdpE
Δcra
ΔfusR
ΔkdpE Δcra
ΔfusR Δcra
ΔfusR ΔkdpE
ΔfusR ΔkdpE Δcra
