# Supplementary material for: Catabolite and Oxygen Regulation of Enterohemorrhagic Escherichia coli Virulence
Source: mBio. 2016 Nov 22;7(6):e01852-16. doi: 10.1128/mBio.01852-16 (PMC5120142; doi:10.1128/mBio.01852-16)
Supplement: Table S1 — Strains and plasmids used in this study. [file mbo006163086st1.docx]

Supplemental Table 1. Strains and plasmids

| Strain | Genotype | Reference |
| --- | --- | --- |
| 86-24 | Stx^+^ EHEC strain serotype O157:H7 | Griffin *et al*. 1988 |
| ARP02 | 86-24Δ*fusR* | Pacheco *et al*. 2012 |
| JN01 | 86-24Δ*cra* | Njoroge *et al*. 2012 |
| MMC03 | 86-24Δ*espB* | This study |
| KCB01 | 86-24Δ*kdpE* | This study |
| KCB02 | 86-24Δ*kdpE*Δ*cra* | This study |
| KCB03 | 86-24Δ*fusR*Δ*cra* | This study |
| KCB04 | 86-24Δ*fusR*Δ*kdpE* | This study |
| KCB05 | 86-24Δ*fusR*Δ*kdpE*Δ*cra* | This study |
| DH5α | F^-^ Φ80*lac*ZΔM15 Δ(*lac*ZYA-*arg*F) U169 *rec*A1 *end*A1 *hsd*R17 (rk^-^, mk^+^) *pho*A*sup*E44 λ^-^*thi*^-^1 *gyr*A96 *rel*A1 | Thermo Fisher |
| KCB06 | 86-24Δ*fusR* complemented pACYC184*fusR* (pKCB04) | This study |
| KCB07 | 86-24Δ*cra* complemented pACYC184*cra* (pKCB05) | This study |
| KCB08 | 86-24Δ*kdpE* complemented pACYC184*kdpE* (pKCB06) | This study |
| KCB09 | 86-24Δ*kdpE* Δ*cra* complemented pACYC184*kdpE cra* (pKCB07) | This study |
| KCB10 | 86-24Δ*fusR* Δ*cra* complemented pACYC184*fusR cra* (pKCB08) | This study |
| KCB11 | 86-24Δ*fusR* Δ*kdpE* complemented pACYC184*fusR kdpE* (pKCB09) | This study |
| KCB12 | 86-24Δ*fusR* Δ*kdpE* Δ*cra* complemented pACYC184*fusR kdpE cra* (pKCB10) | This study |
|  |  |  |
| Plasmids |  | Reference |
| pKD3 | λ red template plasmid | Datsenko *et al*. 2000 |
| pKD46 | λ red helper plasmid | Datsenko *et al*. 2000 |
| pCP20 | λ red helper plasmid | Datsenko *et al*. 2000 |
| pACYC184 | Cloning vector | New England Biolabs |
| pCR-Blunt II-TOPO | Cloning vector | Invitrogen |
| pKCB01 | *fusR* in pCR-Blunt II-TOPO | This study |
| pKCB02 | *cra* in pCR-Blunt II-TOPO | This study |
| pKCB03 | *kdpE* in pCR-Blunt II-TOPO | This study |
| pKCB04 | *fusR* under P_cat_ promoter in puc19 | This study |
| pKCB05 | *cra* under P_cat_ promoter in puc19 | This study |
| pKCB06 | *kdpE* under P_cat_ promoter in puc19 | This study |
| pKCB07 | *fusR* under P_cat_ promoter in pACYC184 | This study |
| pKCB08 | *cra* under P_cat_ promoter in pACYC184 | This study |
| pKCB09 | *kdpE* under P_cat_ promoter in pACYC184 | This study |
| pKCB10 | *kdpE cra* under P_cat_ promoter in pACYC184 | This study |
| pKCB11 | *fusR cra* under P_cat_ promoter in pACYC184 | This study |
| pKCB12 | *fusR kdpE* under P_cat_ promoter in pACYC184 | This study |
| pKCB13 | *fusR kdpE cra* under P_cat_ promoter in pACYC184 | This study |
