# Supplementary material for: Catabolite and Oxygen Regulation of Enterohemorrhagic Escherichia coli Virulence
Source: mBio. 2016 Nov 22;7(6):e01852-16. doi: 10.1128/mBio.01852-16 (PMC5120142; doi:10.1128/mBio.01852-16)
Supplement: Table S2 — Oligonucleotides and qRT-PCR primers used in this study. [file mbo006163086st2.docx]

Supplemental Table 2. Primers and qRT-PCR primers

| **Primer** | **Genotype** | **Function** |
| --- | --- | --- |
| Z0463lambdaredP1 | attagataataagagaagaaaagtatgattcgggtagtgctggtggatgaccatgttgtggtgtaggctggagctgcttc | Isogenic mutant construction |
| Z0463lambdaredP2 | tccccaggctagcacaataaacagcaaaatcacccaatgacgaagattgcgcgacattaacatatgaatatcctcctta | Isogenic mutant construction |
| JcraredF | cagtcatggctgtggtgcgtgagcacaattaccacccgaacgccgtggcaggtgtaggctggagctgcttc | Isogenic mutant construction |
| JcraredR | cggtgacgttgagccactgccagcaccgggcactgtaagaagtcgagcagtcatatgaatatcctcctt | Isogenic mutant construction |
| kdpEλRed-F | ttttcgtgttacacttccccagcaaactgcccctgaacttgaagaatttcatgaggatatgtgtaggctggagctgcttc | Isogenic mutant construction |
| kdpEλRed-R | atttggcgcaggtttaataataaattaatcactatttaggcgaatttattgaataaaaatcatatgaatatcctccttag | Isogenic mutant construction |
| KCB topo kdpE FW | ttcgaaccagcaaactgcccctgaacttg | pCR-Blunt II cloning |
| KCB topo kdpE RV | gagtactggcgcaggtttaataataaattaatc | pCR-Blunt II cloning |
| Z0463for | gtcgacgttgattgccagcgccgcgc | pCR-Blunt II cloning |
| Z0463rev | tctagaccgcctgttgaccgttattg | pCR-Blunt II cloning |
| KCB topo cra FW | ctatggtttttacattttacgcaagg | pCR-Blunt II cloning |
| KCB topo cra RV | aagtactctacgcgccagagtgaaattc | pCR-Blunt II cloning |
| puc19 to pcat FW | cgacggccagtgaattcgaggatccgagctcatgtacgccaacttttggcgaaaatg | NEBuilder cloning |
| kdpE to pcat RV | cagtttgctgcaacggtggtatatccagtg | NEBuilder cloning |
| pcat to kdpE FW | accaccgttgcagcaaactgcccctgaa | NEBuilder cloning |
| puc19 to kdpE RV | gaggatccccgggtaccgaggagtactggcgcaggtttaataataaat | NEBuilder cloning |
| puc19 to pcat FW | cgacggccagtgaattcgagccactagatatcaagtcgccaacttttggcgaaaatg | NEBuilder cloning |
| fusR to pcat RV | caacgtcgaccaacggtggtatatccagtg | NEBuilder cloning |
| pcat to fusR FW | accaccgttggtcgacgttgattgccag | NEBuilder cloning |
| puc19 to fusR RV | gaggatccccgggtaccgagtctagaccgcctgttgac | NEBuilder cloning |
| puc19 to pcat FW | cgacggccagtgaattcgaggtacagtactcgatcctcccaacttttggcgaaaatg | NEBuilder cloning |
| cra to pcat RV | gtaaaaaccatagcaacggtggtatatccagtg | NEBuilder cloning |
| pcat to cra FW | accaccgttgctatggtttttacattttacgc | NEBuilder cloning |
| puc19 to cra RV | gaggatccccgggtaccgagaagtactctacgcgccag | NEBuilder cloning |
| M13 RV | gtcatagctgtttcctg | Sequencing |
| p184 to kdpE FW | tgccgggcctcttgcgggatgatccgagctcatgtacg | NEBuilder cloning complement plasmid |
| p184cra to kdpE RV | ctgtacctcggagtactggcgcaggttt | NEBuilder cloning complement plasmid |
| p184kdpE to cra FW | gccagtactccgaggtacagtactcgatcc | NEBuilder cloning complement plasmid |
| p184 to cra RV | atgctgtcggaatggacgataagtactctacgcgccag | NEBuilder cloning complement plasmid |
| p184 to fusR RV | tgtcggaatggacgattctagaccgcctgttgac | NEBuilder cloning complement plasmid |
| p184 to fusR FW | ggcctcttgcgggatcgagccactagatatcaagtc | NEBuilder cloning complement plasmid |
| p184fusR to kdpE RV | tagtggctcggagtactggcgcaggttt | NEBuilder cloning complement plasmid |
| p184kdpE-fusR FW | gccagtactccgagccactagatatcaagtc | NEBuilder cloning complement plasmid |
| p184cra to fusR RV | ctgtacctcgtctagaccgcctgttgac | NEBuilder cloning complement plasmid |
| p184fusR to cra FW | gcggtctagacgaggtacagtactcgatcc | NEBuilder cloning complement plasmid |
|  |  |  |
| **qRT-PCR** | **Genotype** | **Reference** |
| KCB cra RT FW | ctaactatcttgaacgccaggc | qRT-PCR |
| KCB cra RT RV | agcaggcaatcagcagttg | qRT-PCR |
| Z0463F | gcgcaccgcctgtactaact | qRT-PCR |
| Z0463-343R1 | cggtgccagcgggtatt | qRT-PCR |
| kdpE RT FW | tgcatctcaccccgattga | qRT-PCR |
| kdpE RT RV | gcgctgggtgagcacttt | qRT-PCR |
| eae RT forward | gctggccttggtttgatca | qRT-PCR |
| eae RT reverse | cgctcccgagtgcaaaa | qRT-PCR |
| espA RT forward | tcagaatcgcagcctgaaaa | qRT-PCR |
| espA RT reverse | cgaaggatgaggtggttaagct | qRT-PCR |
| rpoA RT forward | gcgctcatcttcttccgaat | qRT-PCR |
| rpoA RT reverse | cgcggtcgtggttatgtg | qRT-PCR |
